# Sex and freezing of gait in Parkinson’s disease: a systematic review and meta-analysis

**DOI:** 10.1007/s00415-020-10117-w

**Published:** 2020-07-30

**Authors:** Anouk Tosserams, Masood Mazaheri, Priya Vart, Bastiaan R. Bloem, Jorik Nonnekes

**Affiliations:** 1 Department of Rehabilitation, Center of Expertise for Parkinson & Movement Disorders, Donders Institute for Brain, Cognition and Behaviour, Radboud University Medical Centre, PO Box 9101, 6500 Nijmegen, HB The Netherlands; 2 Department of Neurology, Center of Expertise for Parkinson & Movement Disorders, Donders Institute for Brain, Cognition and Behaviour, Radboud University Medical Centre, Nijmegen, The Netherlands; 3 School of Sport, Exercise and Rehabilitation Sciences, Center of Precision Rehabilitation for Spinal Pain (CPR Spine), College of Life and Environmental Sciences, University of Birmingham, Birmingham, UK; 4 Department of Health Evidence, Radboud University Medical Center, Nijmegen, The Netherlands; 5 Department of Rehabilitation, Sint Maartenskliniek, Nijmegen, The Netherlands

**Keywords:** Parkinson, FOG, Freezing of gait, Gender, Prevalence

## Abstract

**Objective:**

It is unknown how sex affects the prevalence of freezing of gait (FOG). We conducted a systematic review and meta-analysis to establish the sex-specific prevalence of FOG in persons with Parkinson’s disease (PD). In addition, we investigated whether men and women were represented accurately in intervention trials targeting FOG.

**Methods:**

We queried the EMBASE and PubMed databases and identified 2637 articles. Of these, 16 epidemiological studies were included in the meta-analysis, and 51 intervention studies were included in the comparative analysis.

**Results:**

In total, 5702 persons were included in the final meta-analysis of epidemiological studies. The pooled estimate of overall FOG prevalence was 43% [95% CI 33–53%]. We found no difference in FOG prevalence between men [44% (34–54%)] and women [42% (31–52%)] with PD. However, women were markedly underrepresented in intervention trials targeting FOG, with an average proportion of only 29.6% of women in trial populations. The percentage of women included in trials was similar across intervention types but differed greatly across geographical regions.

**Conclusion:**

Sex is not a predictor of FOG. This could aid clinicians in counseling persons with PD about FOG. Importantly, a global effort is needed to include more women into clinical trials. Given the skewed distribution of men and women included in intervention trials targeting FOG, caution might be warranted when extrapolating results from FOG trials to women.

**Electronic supplementary material:**

The online version of this article (10.1007/s00415-020-10117-w) contains supplementary material, which is available to authorized users.

## Introduction

Freezing of gait (FOG) is a common and disabling phenomenon in people with Parkinson’s disease (PD). It is characterized by brief episodes during which patients experience their feet as being “glued to the floor” [1]. Presence of FOG is an important predictor of future falls and loss of independence, and reduces quality of life of affected individuals [2, 3]. The exact mechanisms underlying FOG are not fully understood, but several factors seem associated, including longer disease duration, cognitive decline and presence of depression or anxiety [4–7]. Interestingly, most video-illustrated case reports and case series on FOG display footage of men [8–12]. This may suggest that FOG is more common in men compared to women. However, it remains unclear whether sex affects the prevalence of FOG [13, 14]. Gaining more insight into potential sex differences in FOG prevalence could aid clinicians in counselling PD patients, as well as researchers in selecting an appropriate study population for clinical trials targeting FOG. In this systematic review and meta-analysis, we report pooled estimates of the sex-specific prevalence of FOG in persons with PD. We also investigate whether this sex distribution is adequately reflected in recent clinical trials targeting FOG.

## Methods

### Inclusion criteria

Main selection criteria and methods of analysis were specified and documented in advance. The systematic review was conducted in accordance with the PRISMA statement [15], following an a priori protocol (available upon request). The criteria for eligibility are reported in Table 1. Key criteria for epidemiological studies included: observational cohort- or cross-sectional study design, ambulatory, outpatient or community-based setting, and a minimum of 100 male and female participants with FOG included. Key criteria for intervention studies included: intervention studies published between June 2014 and December 2019, ambulatory, outpatient or community-based setting, and a minimum of 10 participants with FOG included.

**Table 1:** Criteria for eligibility, per arm of the systematic review

Epidemiological studies	Intervention studies
Inclusion criteria	Inclusion criteria
Includes human participants diagnosed with idiopathic PD;	Includes human participants diagnosed with idiopathic PD and FOG;
Includes both male and female participants;	Includes ≥ 10 participants with FOG in the final analysis;
Includes ≥ 100 participants with FOG in the final analysis;	Reports sex distribution of study participants;
Reports prevalence of FOG within the cohort;	Published between June 2014 – December 2019;
Reports sex distribution within the cohort and within FOG subgroup;	Published in English or Dutch
Observational cohort studies (retrospective or prospective) and cross-sectional studies;	
Ambulatory, outpatient or community-based settings only;	
Published in English or Dutch	

Exclusion criteria	Exclusion criteria
Studies that enroll participants who are receiving a particular intervention;	Inpatient or acute care settings;
Inpatient or other acute care settings;	Interventions specifically targeted to either men, or women (e.g. hormonal therapy in women);
Other studies that cannot be expected to provide generalizable estimates of prevalence	Interventions specifically targeted to a specific subgroup of PD patients with FOG (e.g. DBS populations)

*PD* Parkinson’s disease, *FOG* freezing of gait, *DBS* deep brain stimulation

### Search strategy

In June 2019 the PubMed (NLM) and EMBASE (Elsevier) databases were searched. The search strategy was determined with the help of a medical librarian and was used for the selection of both epidemiological studies and intervention studies. “Freezing of gait”, “Parkinson’s disease” and related terms were used. Full details of the search strategy are available in *Appendix e*-*1*. Reference lists of included studies were examined for additional relevant studies. An update search was conducted in December 2019, to check for relevant studies that were published since the original search.

### Study screening and selection

All records were assessed for eligibility by two independent reviewers (AT, MM). Any disagreement was resolved by a third independent reviewer (JN). Only unique nonoverlapping study populations were included. Reasons for exclusion of studies were logged throughout the process. In- and exclusion criteria were specifically designed to eliminate epidemiological studies with a high risk of bias. No formal quality assessment took place for the intervention studies, since the focus of this review was to investigate the sex distribution within the study sample, and not the actual effect of the intervention.

### Standard protocol approvals, registrations, and patient consents

No ethical approval was required for this systematic review and meta-analysis. The predefined review protocol was submitted to the PROSPERO database (pending).

### Data extraction

Data extraction was performed by two independent reviewers (AT, MM). Any discrepancies were resolved by consulting a third reviewer (JN). All data were recorded in a predefined data extraction form. When necessary, corresponding authors were contacted to provide missing or additional information.

For all included epidemiological studies, the following data were extracted: study location, study setting, primary outcome, manner of recruitment, criteria used to establish presence of FOG, patient characteristics including mean age, mean disease duration, and mean (MDS)-UPDRS part III scores, total participants included, total male participants included, total participants with FOG included, and total male participants with FOG included.

For all intervention studies, the following data were extracted: study location, intervention type (e.g. physiotherapy/cueing, pharmacological), a short summary of the intervention, total participants with FOG included, and total male participants with FOG included.

### Synthesis and statistical analysis

For each epidemiological study, both the overall and the sex-specific prevalence of FOG were calculated. Overall FOG prevalence per study was calculated as follows: (number of participants with FOG/total number of participants in the study) × 100. Sex-specific prevalence of FOG per study was calculated for both men and women as follows: (number of men or women included in the FOG subgroup)/(number of men or women included in the total sample) × 100.

Meta-analyses were performed to provide pooled estimates of overall FOG prevalence, and FOG prevalence among men and women separately. Analyses were performed in Stata (StataCorp. 2019. Stata Statistical Software: Release 16. College Station, TX: StataCorp LLC.), using the *metaprop* program for pooling binomial data [16]. Additionally, a multivariable meta-regression analysis was performed, to investigate the influence of sex on overall FOG prevalence, independently of disease duration and -severity. A random effects model was employed for all analyses, because of the clinical heterogeneity of included studies. The degree of statistical heterogeneity was assessed using the *I*^*2*^ index. *p* values < 0.05 were considered to be significant.

For each intervention study, sex distribution of included participants was calculated by: (number of men with FOG included/total number of participants with FOG included) × 100. Studies including < 45% male participants were marked as ‘female-predominant’, studies including 45–55% male participants were marked as ‘neutral’, and studies including > 55% male participants were marked as ‘male-predominant’. Studies were categorized per intervention type, and geographical region.

### Data availability

Data are available to qualified investigators on request to the corresponding author.

## Results

### Literature search

**Fig. 1:**
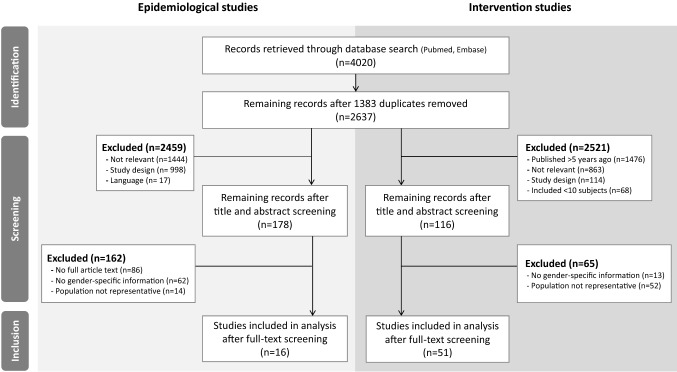
PRISMA flow diagram of the screening and selection process. No full article text (e.g. conference abstracts); Population not representative (e.g. preselected groups such as persons who had all received deep brain stimulation prior to FOG onset)

In total, 2637 deduplicated records were retrieved from PubMed and EMBASE. After title and abstract screening, the full text of 178 articles was evaluated, after which 16 epidemiological reports were included in the final meta-analysis. In addition, 51 recent intervention studies on FOG were included for comparative analyses. Figure 1 shows a flowchart of the screening and selection process. Conference abstracts were screened during the process, but excluded because (1) the information provided was too limited to determine whether the study met all the pre-defined inclusion criteria, or (2) a published final full article was also available, and part of the search result. Corresponding authors had to be contacted for additional information in ten cases. All queries concerned missing patient characteristics data for subgroup analyses (e.g. mean age or UPDRS motor score of the total cohort). One corresponding author responded. After three months, the remaining nine queries were marked as missing data.

### Estimation of sex-specific prevalence of FOG

The 16 epidemiological studies in the final meta-analysis included a total of 5702 persons with PD. The average number of participants per study was 356 (range 100–990). Five studies included more than 500 participants. The average percentage of men included in the studies was 57.5% (range 41.1–66.1%). The mean disease duration of included participants was 7.0 years (range 4.8–12.1). Studies were conducted in Europe (*n *= 6), Asia–Pacific (*n *= 6), and North America (*n *= 4). In most studies, presence of FOG was identified using item 3 of the Freezing of Gait Questionnaire [17] (*n *= 7). In other studies, item 14 of the UPDRS part II [18] (*n *= 3), item 1 of the New Freezing of Gait Questionnaire [19] (*n *= 1), and other self-reported questionnaires (*n *= 3) were used to establish presence of FOG. In one study the presence of FOG was retrospectively extracted from medical records. One study did not report their method used to identify the presence of FOG. The full extracted study data are available in Online Appendix e-2.

**Fig. 2:**
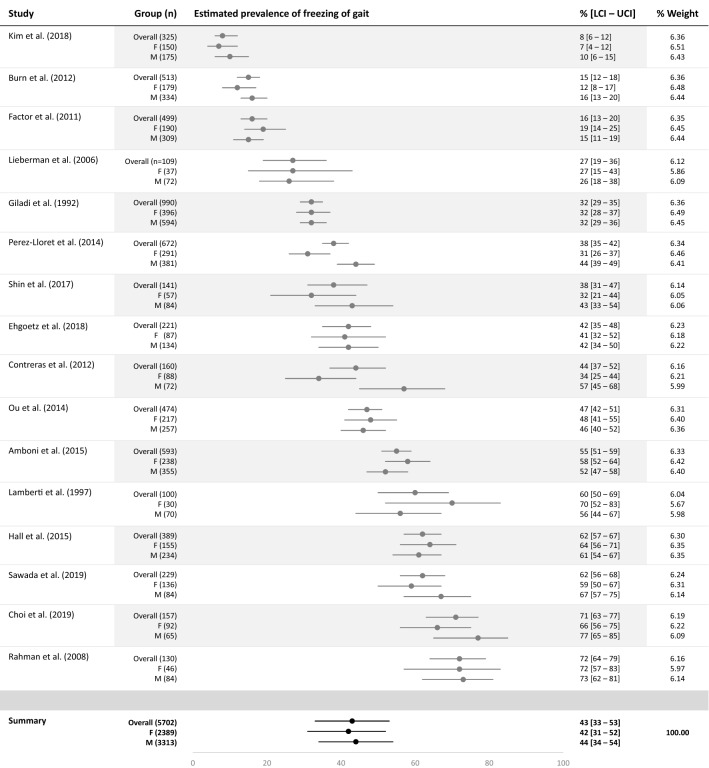
Forest plot of pooled estimates of overall and sex-specific prevalence of freezing of gait in Parkinson’s Disease. *F* female, *M* male, *LCI* lower limit of 95% confidence interval, *UCI* upper limit of 95% confidence interval

Figure 2 presents the forest plots of pooled estimates of overall prevalence of FOG, as well as sex-specific prevalence of FOG. The pooled estimate of overall prevalence of FOG was 43% (95% CI 33–53%). The pooled estimate of FOG prevalence for men was 44% (95% CI 34–54%), and for women 42% (95% CI 31–52%). Included studies were highly heterogeneous (*I*^*2*^> 97%).

A multivariate random effects meta-regression did not demonstrate a relationship between sex and overall FOG prevalence (*p *= 0.333).

### Sex distribution in recent intervention trials on FOG

A total of 51 intervention studies were included in the comparative analysis. Included studies were categorized per intervention type: physiotherapy/cueing (*n *= 32), non-invasive brain stimulation (*n *= 9), pharmacological treatment (*n *= 5), neurosurgical intervention (*n *= 4), or cognitive training (*n *= 1). Most studies were performed in Europe (*n *= 23), followed by Asia (*n *= 10), and North America (*n *= 9).

**Fig. 3:**
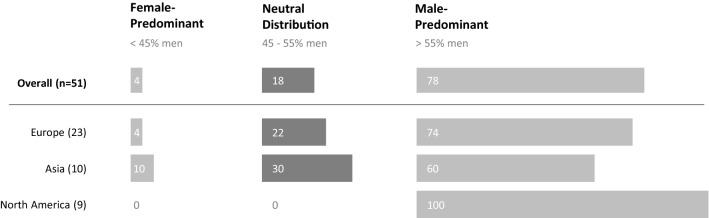
Overall sex distribution (%) in intervention studies on freezing of gait published in the last five years, stratified per geographical location. Due to the limited amount of studies performed in the Middle East (*n *= 2), the Pacific (*n *= 2), South America (*n *= 3), and intercontinental collaborations (*n *= 2), these are not presented as separate subcategories. They are represented in the ‘Overall’ category. All nine studies concerned were male-predominant

The overall sex distribution of included participants is presented in Fig. 3. Out of 51 intervention studies, a mere 9 (17.6%) trials included a neutral sample in terms of sex distribution, whereas 40 (78.4%) trials were male-predominant. On average, 29.6% (range 0–56.7%) of study participants were women. The percentage of women included in trials was similar across intervention types: physiotherapy/cueing (mean: 29%, range 0–57%), non-invasive brain stimulation (mean: 34%, range 15–50%), pharmacological treatment (mean: 30%, range 14–52%), and neurosurgical interventions (mean: 29%, range 8–46%). Moreover, Fig. 3 demonstrates the results stratified by geographical location, where a marked difference in the recruitment of women is apparent across regions. Notably, all trials performed in North America (*n *= 9) were male-predominant.

## Discussion

Our main aim was to establish the sex-specific prevalence of freezing of gait (FOG) in PD. We also investigated whether recent intervention trials targeting FOG portrayed an accurate representation of the FOG disease population in terms of sex distribution. We found no difference in FOG prevalence between men and women with PD. However, women were markedly underrepresented in recent intervention trials targeting FOG.

The absent sex difference in FOG prevalence contradicts previous findings in a large cohort of 6,620 patients, where male sex was identified as a predictor of FOG in PD (OR 1.19 [1.04–1.35]) [13]. This study by Macht et al. was not included in the present meta-analysis because it did not report the sex distribution within the FOG subgroup. An obvious strength of their study was the large number of respondents, and the fact that the included men and women were similar in terms of age and disease duration–the latter features are well-known determinants of FOG prevalence [20]. However, the outcomes of the study by Macht et al. should be interpreted with some caution, for various reasons. First, the study was originally designed to investigate the predictors of sudden onset of sleepiness, and the observed FOG prevalence resulted from post hoc analyses. Second, the definition of freezing that was used differed from validated self-reported questionnaires, such as the NFOG-Q and FOG-Q. Specifically, their definition was not limited to FOG, but encompassed freezing in a broader sense, also including upper limb freezing and freezing of speech. Third, the five possible answer options as to how often respondents experienced freezing were dichotomized. In doing so, respondents who reported freezing less than twice a month were not included in the freezing subgroup, which might have affected the prevalence estimate. In the present meta-analysis, we were able to provide a similar sample size, with enough power to study potential sex differences in FOG prevalence among people with PD, by pooling data from a myriad of smaller studies.

Our results show that the sex distribution in recent intervention trials targeting FOG in PD is skewed towards men by nearly 20%. There are several explanations for this observed difference. First, PD is slightly more prevalent in men compared to women, but this cannot fully explain the difference in inclusion of men and women in FOG trials. In 2016, approximately 6.1 million individuals worldwide had PD, of whom 2.9 million (47.5%) were women and 3.2 million (52.5%) were men [21]. While the age-standardized prevalence of PD is higher in men [21], the lifetime risk of developing PD is 4.4% for men and 3.7% for women [22]. The sex difference we observed in FOG intervention studies was much greater. Second, women might be less likely to be invited to participate in FOG trials, because they may be underrepresented in specialized clinics, which generally initiate such investigations [23]. According to a retrospective observational study investigating the predictors of specialist care utilization, women are less likely to receive neurologist care compared to men [24]. Third, women may theoretically be less inclined to participate in FOG trials, because they might cope differently with their disease [25]. For example, women could be better at self-management, and therefore less likely to seek neurology care. Fourth, women are more likely to experience depression and anxiety [26], which may negatively affect both their interest to partake in clinical trials, as well as their chances to fulfil fit the inclusion criteria. The latter notion could explain why the observed sex gap in FOG research is considerably larger than what was previously noted for PD research as a whole (20% gap in FOG research versus 7% in general PD research) [27], since depression and anxiety are both factors associated with FOG [6, 7]. Finally, regardless of disease-specific reasons, underrepresentation of women in clinical trials appears to be a generic challenge, which is increasingly recognized across other fields of clinical research as well, including cardiology and oncology [28, 29]. A systematic search of nine prominent medical journals regarding randomized controlled trials concluded that the median enrollment of women in the 56 included studies was a mere 37% [30].

As an incidental finding, we found that the inclusion of women in trials differed between study regions. Consistent with a previous study on sex distribution in PD clinical trials, studies conducted in Asia included relatively more women compared to studies conducted in Europe or North America [27]. Examining whether e.g. potential cultural differences in gender roles contribute to this difference in female recruitment is an interesting topic for further investigation.

There are some precautions to take into account when interpreting the results of the present study. First, this review may not be exhaustive due to the limitations of the search strategy. Second, most of the included observational studies identified FOG through self-reported questionnaires. The question therefore remains whether possible differences in the prevalence of FOG were masked by differences in the way men and women might experience and report their motor symptoms. Future work should therefore also focus on patients with FOG that is objectively verified by an experienced examiner. Additionally, cognitive status should be taken into account.

The present finding that sex is not a predictor of FOG could aid clinicians in counselling persons with PD about FOG. Our findings also raise the question whether results from PD trials can be fully extrapolated to women with PD, as women were underrepresented [27]. Future studies may establish the exact impact of this sex data gap, e.g. by investigating whether sex differences affect the efficacy of different FOG interventions. Most importantly, a global effort must be undertaken to include a more representative proportion of women into future clinical trials.

## Electronic supplementary material

Below is the link to the electronic supplementary material.

Supplementary material 1 (DOCX 41 kb)

Supplementary material 2 (DOC 68 kb)
