# Supplementary material for: Sex and freezing of gait in Parkinson’s disease: a systematic review and meta-analysis
Source: J Neurol. 2020 Jul 30;268(1):125–32. doi: 10.1007/s00415-020-10117-w (PMC7815550; doi:10.1007/s00415-020-10117-w)
Supplement: Supplementary file 1 — Supplementary material 1 (DOCX 41 kb) [file 415_2020_10117_MOESM1_ESM.docx]

**Appendices**

**Appendix e-1.** Detailed search strategy ____________________________________________ p. 2

**Appendix e-2.** Extracted study data _______________________________________________ p. 3

**Appendix e-1.** Detailed search strategy

**PubMed database**

((((fog[tiab] OR fogq[tiab] OR freez*[tiab] OR "Freezing Reaction, Cataleptic"[Mesh] OR frozen gait*[tiab]) AND ("Parkinson Disease"[Mesh] OR parkinson*[tiab])))) NOT (animals[Mesh] NOT humans[Mesh])

**EMBASE database**

| 1. | "freezing of gait"/ or catalepsy/ |
| --- | --- |
| 2. | (Fog or fogq or freez* or frozen gait*).ti,ab,kw. |
| 3. | 1 or 2 |
| 4. | Parkinson disease/ |
| 5. | parkinson*.ti,ab,kw. |
| 6. | 4 or 5 |
| 7. | 3 and 6 |
| 8. | limit 7 to "humans only (removes records about animals)" |

**Appendix e-2.** Extracted study data

**Epidemiological studies**

| Study | Title | Location | Outcome | Recruitment | FOG criteria | Mean disease duration cohort (years) | Mean MDS-UPDRS score cohort* | Mean age cohort (years) | Total subjects (n) | Total men  (n (%)) | Total FOG (n) | FOG men (n (%)) |
| --- | --- | --- | --- | --- | --- | --- | --- | --- | --- | --- | --- | --- |
| Amboni et al. 2015 | Prevalence and associated features of self-reported freezing of gait in Parkinson disease: The DEEP FOG study | Italy | Prevalence of self-reported FOG | Consecutive recruitment from outpatient clinic | FOG-Q item-3 ≥ 1 | 8,1 | 31,1 | 66,9 | 593 | 355 (59.9) | 325 | 186 (57.2) |
| Burn et al.  2012 | Parkinson’s Disease Motor Subtypes and Mood | UK | Association between PD motor subtypes and mood | Consecutive recruitment from outpatient clinic | UPDRS part II item 14 > 1 | 5 | 33,4 | 67,9 | 513 | 334 (65.1) | 75 | 54  (72.0) |
| Choi et al.  2019 | Factors associated with freezing of gait in patients with Parkinson’s disease | South Korea | Factors associated with FOG | Consecutive recruitment from outpatient clinic | FOG-Q item-3 ≥ 1 | 6,8 | 33,6 | 69,6 | 157 | 65  (41.1) | 111 | 50  (45.0) |
| Contreras et al.  2012 | Risk factors for freezing of gait in Parkinson's disease | Spain | Risk factors of FOG | Consecutive recruitment from outpatient clinic | FOG-Q item-3 ≥ 1 | 8,1 | 35,8 | 72 | 160 | 72  (45.0) | 71 | 41  (57.7) |
| Ehgoetz et al.  2018 | Predicting the Onset of Freezing of Gait: A Longitudinal Study | Australia | Predictors of FOG | Consecutive recruitment from outpatient clinic | FOG-Q item-3 ≥ 1 | 6,4 | 45 | 68,4 | 221 | 134  (60.6) | 92 | 56  (60.9) |
| Factor et al.  2011 | Postural instability/gait disturbance in Parkinson's disease has distinct subtypes: An exploratory analysis | USA | Predictors of FOG | Through an existing cohort | UPDRS part II item 14 > 1 | 8,5 |  | 67,7 | 499 | 309  (61.9) | 82 | 46  (56.1) |
| Giladi et al.  1992 | Motor blocks in Parkinson's disease | USA | Prevalence of motor blocks | Database | Extracted from medical records | 6,06 |  |  | 990 | 594  (60) | 318 | 191  (60.1) |
| Hall et al.  2015 | Freezing of Gait and its Associations in the Early and Advanced Clinical Motor Stages of Parkinson’s Disease: A Cross-Sectional Study | Australia | Prevalence of and factors associated with FOG | Consecutive recruitment from outpatient clinic | FOG-Q item-3 ≥ 1 | 6,9 | 37,7 | 71,1 | 389 | 234  (61.4) | 241 | 142  (58.9) |
| Kim et al.  2018 | Association of metals with the risk and clinical characteristics of Parkinson's disease | South Korea | Association between serum metal concentration and PD clinical symptoms | Outpatient clinic | Not reported: *"patients were assessed for FOG"* | 7 |  | 65,2 | 325 | 175  (53.8) | 27 | 17  (63.0) |
| Lamberti et al.  1997 | Freezing gait in Parkinson's disease | Italy | FOG prevalence | Consecutive recruitment from outpatient clinic | Self-reported questionnaire | 6,5 |  | 61,1 | 100 | 70  (70.0) | 60 | 39  (65.0) |
| Lieberman et al.  2006 | Are Freezing of Gait (FOG) and panic related? | USA | Association between panic and FOG | Consecutive recruitment from outpatient clinic | FOG-Q item-3 and UPDRS II item 14 | 6,4 |  | 69 | 109 | 72  (66.1) | 29 | 19  (65.5) |
| Ou et al.  2014 | Freezing of Gait in Chinese patients with Parkinson Disease | China | Prevalence of FOG | Consecutive recruitment from outpatient clinic | FOG-Q item-3 ≥ 1 | 4,8 | 36,6 | 62,1 | 474 | 257  (54.2) | 221 | 117  (52.9) |
| Perez-Lloret et al.  2014 | Prevalence, Determinants, and Effect on Quality of Life of Freezing of Gait in Parkinson Disease | France | Prevalence of FOG | Outpatient clinic | UPDRS II item 14 |  |  |  | 672 | 381  (56.7) | 257 | 166  (64.6) |
| Study | **Title** | **Location** | **Outcome** | **Recruitment** | **FOG criteria** | **Mean disease duration cohort (years)** | **Mean MDS-UPDRS score cohort*** | **Mean age cohort (years)** | **Total subjects (n)** | **Total men**  **(n (%))** | **Total FOG (n)** | **FOG men (n (%))** |
| Rahman et al.  2008 | The factors that induce or overcome freezing of gait in Parkinson's disease | UK | Factors that induce or overcome FOG | Outpatient clinic, advertisement in newsletter of Parkinson's Disease Society | Gait and Falls Questionnaire (GFQ) | 12,1 |  | 66,7 | 130 | 84  (64.6) | 94 | 61  (64.9) |
| Sawada et al.  2019 | Clinical features of freezing of gait in Parkinson's disease patients | Japan | Clinical features of FOG | Consecutive recruitment from outpatient clinic | NFOG-Q non-zero score |  |  |  | 229 | 93  (40.6) | 142 | 62  (43.7) |
| Shin et al.  2017 | Self-Reported Symptoms of Parkinson's Disease by Sex and Disease Duration | USA | Prevalence of symptoms by sex and disease duration | Michael J Fox foundation | Self-reported, FOG: yes/no | 6,3 |  | 69,7 | 141 | 84  (59.6) | 54 | 36  (66.7) |

* In case the UPDRS was performed, scores were converted by adding 7 points *(Hentz, J.G., et al., Simplified conversion method for unified Parkinson's disease rating scale motor examinations. Mov Disord 2015)*

**Intervention studies**

| Study | Title | Location | Intervention type | Short summary of the intervention | Total subjects with FOG  (n) | Total men with FOG  (n (%)) |
| --- | --- | --- | --- | --- | --- | --- |
| Aggarwal et al.  2019 | Clinical outcomes of step synchronized vibration training in Parkinson's disease patients | Asia | Physiotherapy / cueing | step-synchronized vibtation training using the PDShoe | 17 | 13 (76.5) |
| Agosta et al.  2017 | Brain plasticity in Parkinson's disease with freezing of gait induced by action observation training | Europe | Physiotherapy / cueing | 4-week action observation training | 25 | 18 (72.0) |
| Ahn et al.  2017 | Smart Gait-Aid Glasses for Parkinson's Disease Patients | Asia | Physiotherapy / cueing | Cueing with Smart Gait-Aid glasses | 10 | 7 (70.0) |
| Amini et al.  2018 | Kinect4FOG: monitoring and improving mobility in people with Parkinson’s using a novel system incorporating the Microsoft Kinect v2 | Europe | Physiotherapy / cueing | Kinect laser cueing | 15 | 12 (80.0) |
| Barthel et al.  2018 | The laser shoes: A new ambulatory device to alleviate freezing of gait in Parkinson disease | Europe | Physiotherapy / cueing | Laser shoes cueing | 19 | 16 (84.2) |
| Beck et al.  2015 | Freezing of Gait in Parkinson's Disease: An Overload Problem? | Northern America | Physiotherapy / cueing | Visual cueing | 20 | 18 (90.0) |
| Chang et al.  2017 | Effect of Dual-Mode and Dual-Site Noninvasive Brain Stimulation on Freezing of Gait in Patients With Parkinson Disease | Asia | Non-invasive brain stimulation | tDCS as add-on to rTMS | 32 | 20 (62.5) |
| Cibulcik et al.  2016 | Effects of rasagiline on freezing of gait in Parkinson's disease - an open-label, multicenter study | Europe | Pharmacological | Rasagiline | 40 | 22 (55.0) |
| Clerici et al.  2019 | Land Plus Aquatic Therapy Versus Land-Based Rehabilitation Alone for the Treatment of Freezing of Gait in Parkinson Disease: A Randomized, Controlled Study | Europe | Physiotherapy / cueing | Aquatic therapy as add-on to land-based rehabilitation | 52 | 39 (75.0) |
| Dagan et al.  2018 | Multitarget transcranial direct current stimulation for freezing of gait in Parkinson's disease | Middle East | Non-invasive brain stimulation | tDCS | 20 | 17 (85.0) |
| Delval et al.  2014 | Auditory cueing of gait initiation in Parkinson's disease patients with freezing of gait | Europe | Physiotherapy / cueing | Auditory cueing for gait initiation | 30 | 13 (43.3) |
| Fietzek et al.  2014 | Randomized cross-over trial to investigate the efficacy of a two-week physiotherapy programme with repetitive exercises of cueing to reduce the severity of freezing of gait in patients with Parkinson's disease | Europe | Physiotherapy / cueing | 2-week program of cueing exercises with a physiotherapist | 22 | 16 (72.7) |
| Ginis et al.  2017 | External input for gait in people with Parkinson's disease with and without freezing of gait: One size does not fit all | Europe | Physiotherapy / cueing | Auditory cueing | 15 | 14 (93.3) |
| Iijima et al.  2019 | Efficacy of istradefylline for gait disorders with freezing of gait in Parkinson's disease: A single-arm, open-label, prospective, multicenter study. | Asia | Pharmacological | Istradefylline | 31 | 15 (48.4) |
| Janeh et al.  2019 | Gait Training in Virtual Reality: Short-Term Effects of Different Virtual Manipulation Techniques in Parkinson's Disease | Europe | Physiotherapy / cueing | VR-based gait manipulation strategy | 15 | 15 (100) |
| Janssen et al.  2017 | Cerebellar theta burst stimulation does not improve freezing of gait in patients with Parkinson’s disease | Europe | Non-invasive brain stimulation | Theta burst stimulation to the cerebellum | 17 | 13 (76.5) |
| Janssen et al.  2017 | Usability of Three-dimensional augmented Visual cues Delivered by smart glasses on (Freezing of) gait in Parkinson’s Disease | Europe | Physiotherapy / cueing | Visual cues through smart glasses | 25 | 19 (76.0) |
| Study | **Title** | **Location** | **Intervention type** | **Short description of the intervention** | **Total subjects with FOG (n)** | **Total men with FOG (n)** |
| Jia et al.  2017 | A novel paradigm of variable frequency deep brain stimulation to improve freezing of gait in Parkinson's disease | Asia | Neurosurgical / DBS | Combining high and low frequency stimulation in varying patterns | 28 | 16 (57.1) |
| Kim et al.  2015 | Efficacy of cumulative high-frequency rTMS on freezing of gait in Parkinson's disease | Asia | Non-invasive brain stimulation | Cumulative high-frequency rTMS | 17 | 11 (64.7) |
| Kim et al.  2018 | Stimulation in Supplementary Motor Area Versus Motor Cortex for Freezing of Gait in Parkinson's Disease | Asia | Non-invasive brain stimulation | rTMA in SMA | 12 | 6 (50.0) |
| Kim et al.  2019 | Long-term effect of subthalamic nucleus deep brain stimulation on freezing of gait in Parkinson’s disease | Asia | Neurosurgical / DBS | Long-term effects of STN-DBS | 28 | 15 (53.6) |
| Kleiner et al.  2018 | Automated Mechanical Peripheral Stimulation Effects on Gait Variability in Individuals With Parkinson Disease and Freezing of Gait: A Double-Blind, Randomized Controlled Trial | South America | Physiotherapy / cueing | Tactile cueing (automated mechanical peripheral stimulation effects) | 30 | 21 (70.0) |
| Lu et al.  2017 | Effect of Cue Timing and Modality on Gait Initiation in Parkinson Disease With Freezing of Gait | Northern America | Physiotherapy / cueing | Acoustic, visual and vibrotactile cues | 11 | 7 (63.6) |
| Lu et al.  2018 | The effects of anodal tDCS over the supplementary motor area on gait initiation in Parkinson’s disease with freezing of gait: a pilot study | Northern America | Non-invasive brain stimulation | Anodal tDCS over SMA | 10 | 7 (70.0) |
| Luca et al.  2017 | Dalfampridine in Parkinson's disease related gait dysfunction: A randomized double blind trial | Northern America | Pharmacological | Dalfampridine | 20 | 16 (80.0) |
| Mancini et al.  2018 | Assessment of the ability of open- and closed-loop cueing to improve turning and freezing in people with Parkinson's disease | Northern America | Physiotherapy / cueing | Auditory and tactile cues | 25 | 19 (76.0) |
| Martin et al.  2015 | A Randomized Controlled Feasibility Trial of a Specific Cueing Program for Falls Management in Persons With Parkinson Disease and Freezing of Gait." | Pacific | Physiotherapy / cueing | 6-month standardized, home-based, cueing exercise and education program | 21 | 13 (61.9) |
| McCandless et al.  2016 | Effect of three cueing devices for people with Parkinson's disease with gait initiation difficulties | Europe | Physiotherapy / cueing | Acoustic, visual and vibrotactile cues | 20 | 14 (70.0) |
| Mezzarobba et al.  2017 | Action Observation Plus Sonification. A Novel Therapeutic Protocol for Parkinson's Patient with Freezing of Gait | Europe | Physiotherapy /cueing | Action observation + sonification vs cueing | 22 | 14 (63.6) |
| Myers et al.  2018 | Effects of exercise on gait and motor imagery in people with Parkinson disease and freezing of gait | Northern America | Physiotherapy / cueing | Exercise intervention | 13 | 10 (76.9) |
| Nuic et al.  2018 | The feasibility and positive effects of a customised videogame rehabilitation programme for freezing of gait and falls in Parkinson's disease patients: a pilot study | Europe | Physiotherapy / cueing | Customised videogame rehabilitation programme | 10 | 5 (50.0) |
| Pelosin et al.  2018 | Effect of group-based rehabilitation combining action observation with physiotherapy on freezing of gait in Parkinson’s disease | Europe | Physiotherapy / cueing | Group-based rehabilitation combining action observation training with physical training | 64 | 31 (48.4) |
| Pereira et al.  2016 | Freezing of gait in Parkinson's disease: Evidence of sensory rather than attentional mechanisms through muscle vibration | Intercontinental collaboration | Physiotherapy / cueing | Vibrotactile cues | 16 | 14 (87.5) |

| Study | Title | Location | Intervention type | Short description of the intervention | Total subjects with FOG  (n) | Total men with FOG  (n (%)) |
| --- | --- | --- | --- | --- | --- | --- |
| Pinto et al.  2018 | Automated Mechanical Peripheral Stimulation Improves Gait Parameters in Subjects With Parkinson Disease and Freezing of Gait: A Randomized Clinical Trial | South America | Physiotherapy / cueing | Tactile cueing (automated mechanical peripheral stimulation) | 30 | 21 (70.0) |
| Plotnik et al.  2014 | A motor learning-based intervention to ameliorate freezing of gait in subjects with Parkinson's disease. | Middle East | Physiotherapy / cueing | 6 week progressive motor learning program | 15 | 11 (73.3) |
| Prusch et al.  2018 | Automated mechanical peripheral stimulation and postural control in subjects with Parkinson’s disease and freezing of gait: a randomized controlled trial | South America | Physiotherapy / cueing | Tactile cueing (automated mechanical peripheral stimulation) | 33 | 24 (72.7) |
| Putzolu et al.  2018 | Anodal tDCS over prefrontal cortex improves dual-task walking in Parkinsonian patients with freezing | Europe | Non-invasive brain stimulation | Anodal tDCS over prefrontal cortex | 10 | 6 (60.0) |
| Rahimi et al.  2016 | Patterns and predictors of freezing of gait improvement following rasagiline therapy: A pilot study | Intercontinental collaboration | Pharmacological | Rasagiline | 14 | 12 (85.7) |
| Revuelta et al.  2015 | Pilot study of atomoxetine in patients with Parkinson's disease and dopa-unresponsive Freezing of Gait | Northern America | Pharmacological | Atomoxetine | 10 | 8 (80.0) |
| Santos et al.  2017 | Effects of progressive resistance exercise in akinetic-rigid Parkinson’s disease patients: a randomized controlled trial | Europe | Physiotherapy / cueing | Progressive resistance exercise | 28 | 15 (53.6) |
| Schlenstedt et al.  2018 | Moderate Frequency Resistance and Balance Training Do Not Improve Freezing of Gait in Parkinson's Disease: A Pilot Study | Europe | Physiotherapy / cueing | Moderate Frequency Resistance and Balance Training | 20 | 15 (75.0) |
| Scholten et al.  2017 | Effects of Subthalamic and Nigral Stimulation on Gait Kinematics in Parkinson's Disease | Europe | Neurosurgical / DBS | Subthalamic and nigral stimulation | 12 | 11 (91.7) |
| Smulders et al.  2016 | Effects of a physical rehabilitation program with cognitive challenge for freezing of gait - A pilot study | Northern America | Cognitive training | 6 weeks of Agility Boot Camp exercises with increasing cognitive challenge (C-ABC) and 6 weeks of education (control) | 10 | 10 (100) |
| Stummer et al.  2015 | The walk-bicycle: A new assistive device for Parkinson's patients with freezing of gait? | Europe | Physiotherapy / cueing | Walk-bicycle | 18 | 15 (83.3) |
| Tang et al.  2017 | Rhythmic laser cue is beneficial for improving gait performance and reducing freezing of turning in parkinson’s disease patients with freezing of gait | Asia | Physiotherapy / cueing | Visual cueing | 23 | 10 (43.5) |
| Tard et al.  2016 | Single session intermittent theta-burst stimulation on the left premotor cortex does not alleviate freezing of gait in Parkinson's disease | Europe | Non-invasive brain stimulation | Single session intermittent theta-burst stimulation on the left premotor cortex | 15 | 11 (73.3) |
| Valentino et al.  2014 | Transcranial direct current stimulation for treatment of freezing of gait: a cross-over study | Europe | Non-invasive brain stimulation | tDCS of primary motor cortex | 10 | 5 (50.0) |
| Walton et al.  2018 | Cognitive training for freezing of gait in Parkinson's disease: a randomized controlled trial | Pacific | Cognitive training | 7-week twice-weekly cognitive training | 38 | 25 (65.8) |
| Xie et al.  2018 | Long-term effect of low frequency stimulation of STN on dysphagia, freezing of gait and other motor symptoms in PD | Northern America | Neurosurgical / DBS | low frequency stimulation of STN | 11 | 9 (81.8) |
| Yang et al.  2016 | Immediate Effects of Clock-Turn Strategy on the Pattern and Performance of Narrow Turning in Persons With Parkinson Disease | Asia | Physiotherapy / cueing | Clock-turn strategy during turning | 25 | 13 (52.0) |
| Zhao et al.  2016 | Feasibility of external rhythmic cueing with the Google Glass for improving gait in people with Parkinson's disease | Europe | Physiotherapy / cueing | External rhythmic cueing with the Google Glass | 12 | 9 (75.0) |
